# Supplementary figures and images for: SOX2-OT Binds with ILF3 to Promote Head and Neck Cancer Progression by Modulating Crosstalk between STAT3 and TGF-β Signaling
Source: Cancers (Basel). 2023 Dec 8;15(24):5766. doi: 10.3390/cancers15245766 (PMC10742126; doi:10.3390/cancers15245766)

Figure S1: The whole Western blot figures

Figure 6A

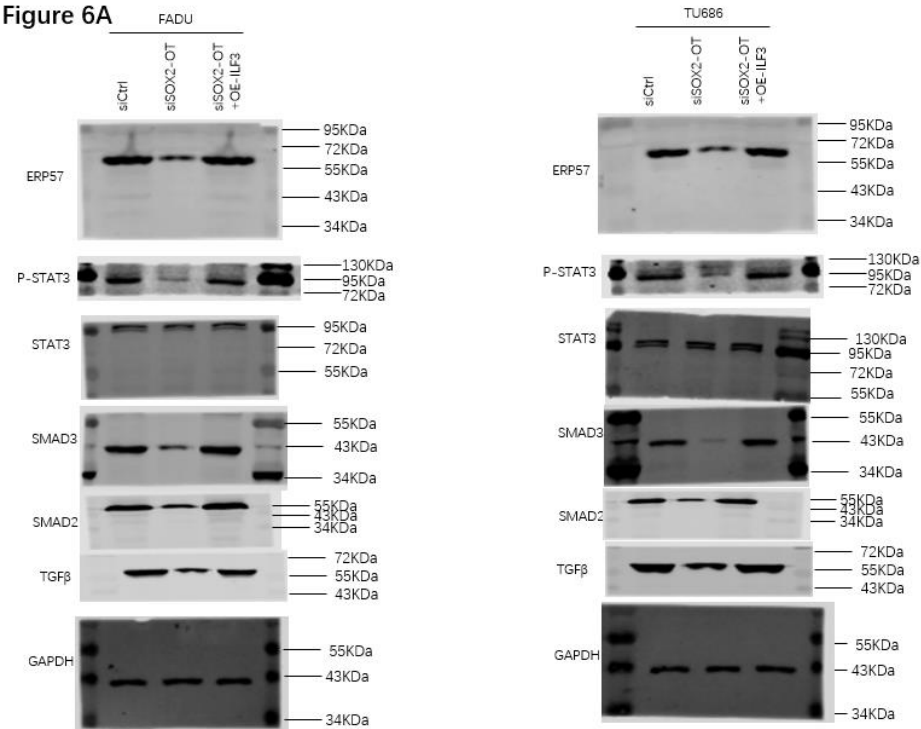

Figure 6B

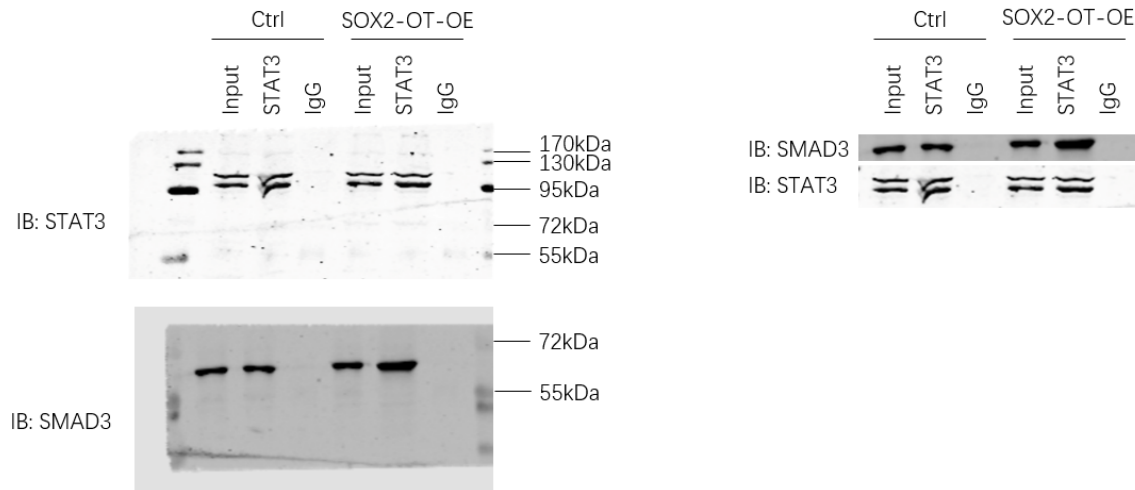

Supplement: Supplementary file 1 [file cancers-15-05766-s001.zip › cancers-2715513-supplementary material/cancers-2715513-Figure S1.pdf]
